# Impact of skin tone on target size detectability in photoacoustic breast imaging

**DOI:** 10.1117/1.BIOS.2.1.012502

**Published:** 2024-11-14

**Authors:** Rhea D. Rasquinha, Mardava R. Gubbi, Muyinatu A. Lediju Bell

**Affiliations:** a Brown University, Department of Biomedical Engineering, Providence, Rhode Island, United States; b Johns Hopkins University, Department of Electrical and Computer Engineering, Baltimore, Maryland, United States; c Johns Hopkins University, Department of Biomedical Engineering, Baltimore, Maryland, United States; d Johns Hopkins University, Department of Computer Science, Baltimore, Maryland, United States; e Johns Hopkins Medicine, Department of Oncology, Baltimore, Maryland, United States

**Keywords:** breast imaging, photoacoustic imaging, acoustic clutter, target visibility, coherence-based beamforming

## Abstract

**Significance:**

Photoacoustic imaging has the potential to improve non-invasive breast cancer diagnosis. However, illumination through the skin introduces a skin tone bias, as greater melanin content increases optical absorption and can create acoustic clutter, reducing the visibility of various target sizes.

**Aim:**

We investigate the impact of skin tone bias as a function of target sizes in three photoacoustic image reconstruction methods: fast Fourier transform (FFT)-based reconstruction, delay-and-sum (DAS) beamforming, and short-lag spatial coherence (SLSC) beamforming.

**Methods:**

The three imaging methods were applied to channel data from multidomain simulations with 757, 800, and 1064 nm wavelengths, 11 target sizes (ranging 0.5 to 3 mm), 18 skin tones [ranging individual typology angles (ITAs), of –54 deg to 60 deg], and a previously validated realistic 3D breast model. The signal-to-noise ratio (SNR) and generalized contrast-to-noise ratio (gCNR) were measured to assess visibility.

**Results:**

With 757 and 800 nm wavelengths, targets underlying dark skin tones (ITA range: −54  deg to −33  deg) with sizes ≤3  mm were poorly visible with ≤2.38 to 4.21 mean SNR and ≤0.46 to 0.74 mean gCNR, with smaller targets generally being more visible with lighter skin tones. A 1064-nm transmit wavelength improved visualization with DAS and SLSC beamforming, relative to both FFT reconstruction with a 1064 nm wavelength and DAS or SLSC beamforming with 757 and 800 nm wavelengths. When combined with SLSC beamforming, the 1064-nm wavelength offered the greatest improvements, enabling visualization of simulated target sizes ranging from 0.5 to 3 mm underlying very light (ITA = 60 deg) to dark (ITA=−54  deg) skin tones, with mean SNR ≤10.01 and mean gCNR ≤1. Visualization of simulated vessel structures derived from *in vivo* photoacoustic images was consistent with simulation-based target size expectations.

**Conclusions:**

Results are promising for advancing next-generation photoacoustic imaging systems for breast cancer diagnosis across the range of skin tones represented in healthcare systems throughout the world.

Statement of Discovery This work provides the first known *in silico* demonstrations to support the benefits of 1064 nm transmit wavelength combined with short-lag spatial coherence (SLSC) beamforming when visualizing target sizes ranging 0.5–3 mm underlying very light (ITA = 60 deg) to dark (ITA = –54 deg) skin tones.

## 1 Introduction

Photoacoustic imaging is an emerging modality for non-invasive screening and detection of breast cancer.^1^ Photoacoustic imaging is based on the photoacoustic effect, by which optical energy is converted to acoustic energy.^2^ Biological tissues absorb energy from a laser light source which causes thermal expansion and the emission of acoustic waves^3^ that can be detected by conventional clinical ultrasound transducers and used to reconstruct an image.^4^ X-ray mammography is the current standard for routine breast cancer screening, but the sensitivity of this imaging modality decreases in patients with greater breast density.^5^^,^^6^ Alternative screening methods include magnetic resonance imaging and ultrasound imaging, but these are limited by high cost and a high false positive rate, respectively.^1^^,^^7^^,^^8^ As the scattering of acoustic waves in tissue is significantly less than that of optical waves,^9^ photoacoustic imaging can be used to generate high-resolution images of hemoglobin content, which is linked to the presence of cancer.

Hemoglobin has high optical absorption in the near-infrared range relative to water and other tissues,^1^ which enables photoacoustic imaging systems to use this wavelength range to detect vascular structures. Tumor angiogenesis is a known indication of malignancy,^10^ and other reported indicators include hypoxia^11^ and elevated hemoglobin levels.^12^ Several photoacoustic imaging systems have been developed to differentiate between the vasculature of benign and malignant breast lesions.^3^ Abeyakoon et al.^13^ developed a feature set based on combined photoacoustic-ultrasound images that improved diagnostic accuracy of malignant lesions. However, these diagnostic vessels vary in size.^13^^–^^17^

To non-invasively image these vascular structures, tissue illumination through the skin is required,^18^ yet it is limited by the high optical absorption coefficient of skin in the visible and near-infrared range due to the presence of melanin,^19^^,^^20^ which decreases optical penetration depths, particularly for vessels located deeper in the tissue. This high optical absorption can also result in acoustic wave emission from the skin into the tissue below, which are reflected by typical ultrasound scatterers and create artifacts and acoustic clutter, reducing image quality.^21^^,^^22^ As skin optical absorption coefficients increase with greater melanin content,^20^ these two limitations significantly compromise photoacoustic image quality for individuals with darker skin tones, creating a skin tone bias in photoacoustic imaging systems.^22^^–^^24^ In addition, Fernandes et al.^25^ confirmed skin as the primary source of clutter in photoacoustic images of a 3D breast tissue model. However, the relationship between skin tone and target size detectability in photoacoustic imaging is currently unknown.

Initially developed to predict the risk of sunburn and skin cancer,^26^ the Fitzpatrick skin type scale has been widely used in dermatology to classify skin tone,^27^ although this scale is limited by inaccuracy and inconsistent use.^28^ The individual typology angle (ITA) provides a more objective evaluation that can be calculated based on the Commission Internationale de l’Eclairage $L^{*}a^{*}b^{*}$ color system.^29^ ITA is also reported to be highly correlated with melanin content in the skin^30^^,^^31^ and is related to the skin optical absorption coefficient and wavelength through the equation proposed by Junior et al.^32^

Short-lag spatial coherence (SLSC) beamforming is an image reconstruction method shown to reduce clutter and improve photoacoustic image quality in noisy environments with low optical fluence.^33^^,^^34^ The SLSC beamformer computes and sums the spatial coherence between signals received at different transducer elements across the short spatial lag region to construct an image.^35^ Clutter sources have low spatial coherence, which enables the mitigation of clutter artifacts in SLSC images.^36^^–^^38^ Fernandes et al.^34^ demonstrated a skin tone bias in photoacoustic imaging of the radial artery in human volunteers, which was successfully mitigated by SLSC beamforming, allowing for clear radial artery visualization in skin tones ranging from light to dark. We expect that SLSC beamforming will similarly improve target and vessel visualization in photoacoustic images used to determine the presence of breast cancer when considering a range of vessel sizes in both lighter and darker skin tones.

In this paper, the impact of skin tone on target size detectability in photoacoustic breast imaging is investigated with multidomain simulations implemented using a previously validated realistic 3D breast model.^39^ Representative target sizes, skin tones, and wavelengths of interest were simulated to quantify target visibility for a range of imaging scenarios with two amplitude-based beamforming methods for comparison with SLSC beamforming. We additionally simulated vessels derived from previously published features of interest (i.e., splayed vessel and claw features^13^) and then compared results with existing *in vivo* data.

## 2 Methods

**Fig. 1:**
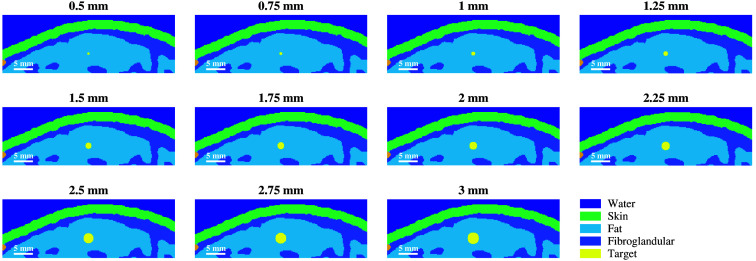
Cross-sectional view of the simulated volumes within the imaging plane, demonstrating various tissue components and ranges of simulated photoacoustic target sizes.

Eleven simulation volumes were constructed based on a realistic 3D breast model immersed in water, with breast tissue classified as extremely dense.^39^ The model volumes were 45×15×20  mm in the lateral, axial, and elevation directions, respectively, with a voxel size of 100  μm in each dimension. In each volume, a spherical photoacoustic target with diameters ranging from 0.5 to 3 mm was generated, in 0.25 increments. This range was chosen based on measurements of blood vessels and other diagnostic features from *in vivo* patient photoacoustic images,^13^^–^^17^ and the optical and acoustic properties of the targets were set to those of blood, which is an important component of tumor detection.^10^^–^^13^ The targets were centered in the lateral and elevation dimensions of the model, and the center of each target was placed at a depth of 8.6 mm from the skin surface (10 mm from the top surface of the volume in the axial direction), as shown in Fig. 1.

Optical simulations were conducted using MCXLAB^40^ to compute the fluence of light in the volumes. A collimated Gaussian beam with a 4-mm waist radius (i.e., $50.3\;{\mathrm{mm}}^{2}$ illumination area) emitting $10^{8}$ photons was centered in the lateral and elevation dimensions and placed at the top of the volume. Although the simulated fluence is reported as normalized values, any incident energy divided by the illumination area is expected to provide information about the fluence that can be expected in a real system. To assess the impact of skin tone on target size detectability, 18 ITA values were simulated (ranging from 60 deg to −54 deg, including three values per skin tone category, as defined in Refs. 29, 41, and 42 and summarized in Table 1). These ITA values correspond to previously reported melanin content-to-basal length ratios ranging from $∼0\;{\mathrm{mm}}^{2}/\mathrm{mm}$ at an ITA of 60 deg to $∼2.1\;{\mathrm{mm}}^{2}/\mathrm{mm}$ at an ITA of −60 deg,^43^ melanosomes/$\mu {\text{m}}^{2}$ ranging $∼20\;\mu m^{-2}$ at an ITA of −36.1 deg to $∼100\;\mu m^{-2}$ at an ITA of 57.4 deg,^43^ and melanin volume fractions (v/v) ranging from 3.6% at an ITA of 56 deg to 40% at an ITA of −47 deg.^23^ We additionally acknowledge that although ITA quantifies perceived skin tone, this standalone metric does not include information about associated molecular or physiological origins (e.g., whether the skin tone perception is due to increases in melanin, blood flow, or veins near the skin surface),^44^ and a recent review article recommends the establishment of an effective melanosome volume fraction parameter based on an assumed epidermal thickness, which can otherwise vary when simply reporting the volume fraction.^45^ For each simulated target size and skin tone, three wavelengths were simulated (i.e., 757, 800, and 1064 nm). These wavelengths were chosen because they are used in existing commercial photoacoustic imaging systems.^3^^,^^13^^,^^15^^,^^46^

**Table 1:** Skin tone categories, corresponding ITA values,^29^^,^^41^^,^^42^ and specific ITA values simulated.

Skin tone category	ITA (deg)	Simulated ITA (deg)
Very Light	>55	60, 58, 56
Light	>41 and ≤55	50, 47, 43
Intermediate	>28 and ≤41	38, 35, 30
Tan	>10 and ≤28	26, 20, 12
Brown	≥−30 and ≤10	5, −10, −20
Dark	<−30	−33, −45, −54

The optical absorption coefficients of water,^47^ blood,^47^ fat,^39^ and fibroglandular tissue^39^ were set based on reported values, and the optical absorption coefficients of skin were calculated using the equation proposed by Junior et al.^32^ The optical scattering coefficients were similarly set based on reported values,^20^ and standardized anisotropy factor and index of refraction values of 0.9 and 1.37, respectively, were used. The optical properties of each tissue component^20^^,^^32^^,^^39^^,^^47^ used in the simulations are summarized in Table 2.

**Table 2:** Optical properties of tissue components.^20^^,^^32^^,^^39^^,^^47^

Component	Optical absorption coefficient (${\mathrm{mm}}^{-1}$)	Optical scattering coefficient (${\mathrm{mm}}^{-1}$)
Water	0.0029 (757 nm) 0.0022 (800 nm) 0.0144 (1064 nm)	1
Skin	Calculated for each wavelength and ITA value using:^32^ $\mu_{a}=45.4\cdot 10^{-0.004\cdot \lambda }\cdot 10^{-0.014\cdot \mathrm{ITA}}$	20.6104 (757 nm) 19.0949 (800 nm) 13.6070 (1064 nm)
Fat	0.005	8.5436 (757 nm) 8.3016 (800 nm) 7.1575 (1064 nm)
Fibroglandular	0.004	13.0649 (757 nm) 11.8491 (800 nm) 7.1567 (1064 nm)
Target (i.e., blood)	0.3042 (757 nm) 0.4370 (800 nm) 0.5484 (1064 nm)	8.5436 (757 nm) 8.3016 (800 nm) 7.1575 (1064 nm)

Acoustic simulations were conducted using k-Wave.^48^ The initial pressure was computed as $\Phi (\overset{\to }{r})\cdot \mu_{a}(\overset{\to }{r})\cdot \Gamma$, where $\Phi (\vec{r})$ is the light fluence map derived from the output of the optical simulation, $\mu_{a}(\vec{r})$ is the optical absorption coefficient map, and Γ is the Grüneisen parameter, which was set to 1. The initial pressure was multiplied by a randomly generated binary absorbers map in which 50% of the pixels were absorbers. To record the acoustic pressure, a linear array ultrasound transducer defined with 128 elements, 0.3 mm pitch, and a 7-MHz center frequency was centered at the top of the volume to simulate an Ultrasonix L14-5/38 linear array transducer.^49^^,^^50^ The sampling frequency was 40 MHz, and the simulated acoustic properties of each tissue component^25^^,^^39^ are summarized in Table 3.

**Table 3:** Acoustic properties of tissue components.^25^^,^^39^

Component	Speed of sound (m/s)	Density ($\mathrm{kg}/m^{3}$)	Attenuation (dB/MHz·cm)
Water	1500	1000	0.0025
Skin	1650	1150	0.35
Fat	1470	937	0.60
Fibroglandular	1515	1040	0.75
Target (i.e., blood)	1584	1040	0.20

As some level of background noise is present in most experimental channel data, noise corresponding to a channel signal-to-noise ratio (SNR) of 20 dB was added to the simulated channel data. Photoacoustic images were then reconstructed using three beamforming methods: (i) amplitude-based beamforming using the fast Fourier transform (FFT) reconstruction technique available in k-Wave,^48^ (ii) standard delay-and-sum (DAS) beamforming, and (iii) SLSC beamforming.^35^ A short-lag value M=10 was used for the SLSC beamformed images, and the axial correlation kernel length was 0.270 mm (i.e., seven samples, which are equivalent to one acoustic wavelength). Envelope detection was applied to the outputs of the DAS and SLSC beamforming algorithms. Each photoacoustic image was normalized by its brightest pixel. Prior to image display, the normalized image data from the FFT and DAS beamforming algorithms were log compressed. The log-compressed (FFT, DAS) or linear (SLSC) image data were then displayed with the same dynamic range per image formation method to facilitate qualitative comparisons.

The axial and lateral resolution of the FFT and DAS images, determined with full-width at half maximum (FWHM) measurements of a point target surrounded by water, located at a 10-mm depth, is 0.84 and 0.73 mm for the DAS images and 0.79 and 0.41 mm for the FFT images, respectively. These axial and lateral FWHM values were calculated by averaging the columns and rows, respectively, immediately adjacent to the target center line (i.e., an average of three rows or columns total). The corresponding SLSC lateral FWHM determined with PhocoSpace^33^^,^^51^^,^^52^ and target sizes of 0.5 and 3 mm are 2.11 and 3.16 mm, respectively, when using the same parameters described above (e.g., a Gaussian light profile width of 8 mm, which corresponds to 4-mm waist radius; an M value of 10; an initial pressure distribution SNR of 20 dB; an imaging field depth of 15 mm; a target center depth of 10 mm with background and target optical absorption coefficients of 0.144 and $5.484\;{\mathrm{cm}}^{-1}$, respectively; an absorber density of $5\times 10^{7}\;{\text{absorbers}/m}^{2}$, which corresponds to 50% of pixels being absorbers; and a transducer with 128 elements, 0.3 mm pitch, 4.9 MHz bandwidth, and 7 MHz center frequency). However, it is known that multiple factors can affect SLSC lateral resolution (e.g., light profile size, target size, noise, M value, and correlation kernel size^33^^,^^51^^–^^54^). The SLSC axial resolution is expected to be greater than the axial correlation kernel length^54^ (i.e., >0.270  mm).

To quantify target detectability, the SNR and generalized contrast-to-noise ratio (gCNR)^55^^–^^57^ were calculated from the normalized image data. The SNR was calculated using the equation

$$\mathrm{SNR}=\frac{\mu_{i}}{\sigma_{o}},$$ (1)

where $\mu_{i}$ is the mean signal amplitude (i.e., after envelope detection and/or normalization, prior to any log compression) within the circular region of interest (ROI) inside the target and $\sigma_{o}$ is the standard deviation of signal amplitudes within the annular ROI outside the target. Each ROI was determined once per target size, using FFT images with no added noise, created with the 757 nm wavelength and 60 deg ITA, as these images were the closest to the ground truth. The circular target ROI was the same size as the target size and concentric with the diameter of the target. The annular background ROI was created with an inner radius $\sqrt{2}\cdot r$, where r is the radius of the target ROI, and an outer radius such that the area of the background and the target ROIs were equal. The same ROIs were then replicated for each wavelength, ITA value, and beamforming method investigated, and were applied to the following gCNR equation (which is a discretization of the equation used to calculate gCNR with probability density functions^56^^,^^57^):

$$\mathrm{gCNR}=1-\overset{N-1}{\underset{k=0}{\sum }}\;\min\{h_{i}(x_{k}),h_{o}(x_{k})\},$$ (2)

where N is the number of bins centered at $\left\{x_{0},x_{1},\ldots ,x_{N-1}\right\}$; $h_{i}$ and $h_{o}$ are the histograms computed for the target and background ROIs, respectively; and k is the bin index. A total of N=32 bins were used to create the histograms. These image analyses were performed with MATLAB software (Mathworks, Natick, MA, United States).

**Fig. 2:**
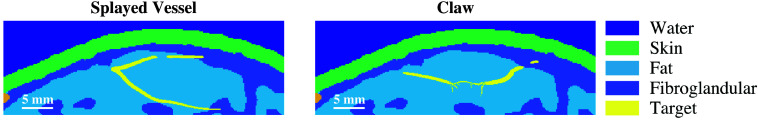
Cross-sectional view of the simulated volumes with splayed vessel and claw structures within the imaging plane.

Although standardized spherical targets are useful in analyzing target detectability limits, clinical photoacoustic targets are more complex in shape and structure. To translate results to more realistic models, two additional simulation volumes were generated with incorporated blood vessel structures based on patient photoacoustic images.^13^ A splayed vessel and claw, which are features of benign and malignant lesions, respectively, were generated in Fusion 360 (Autodesk, San Francisco, CA, United States) and incorporated into the 3D breast model^39^ using MATLAB by creating a triangulation from the exported .stl files. A slice of each volume is shown in Fig. 2. The optical and acoustic properties of the vessel structures were set to those of blood, and the multidomain simulations described above were conducted. Photoacoustic images were generated using the same three beamformers described above (i.e., FFT, DAS, and SLSC).

## 3 Results

### 3.1 Photoacoustic Images of Small Targets

**Fig. 3:**
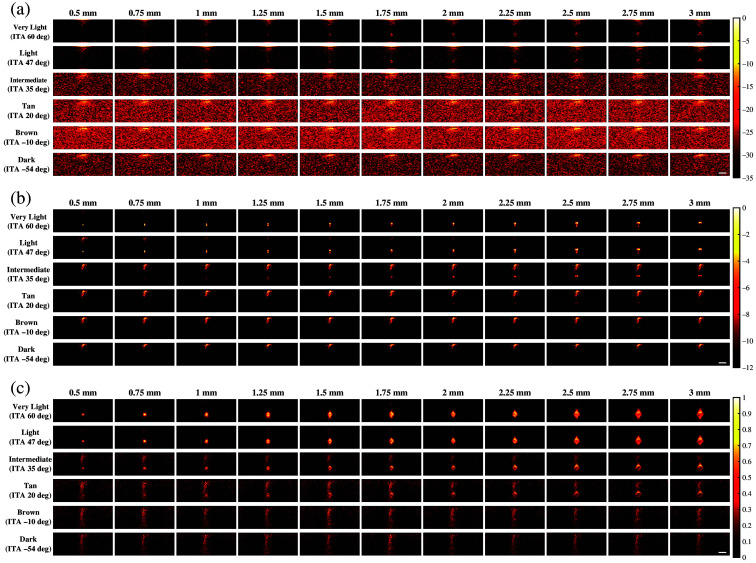
Photoacoustic images created with 757 nm wavelength, multiple skin tones, and (a) FFT, (b) DAS, and (c) SLSC beamforming. The white scale bar represents 5 mm. The skin signal does not appear uniformly across the top of these images because of the size of the light profile (i.e., 4 mm radius, as noted in Sec. 2).

Figure 3 shows representative examples of photoacoustic images generated for simulations using 757 nm wavelength and FFT, DAS, and SLSC beamforming. With FFT beamforming [Fig. 3(a)], targets smaller than 1.75 mm are generally difficult to visualize regardless of skin tone. In addition, for very light (ITA=60  deg) and light (ITA=47  deg) skin tones, target visibility generally increases as the target size increases. However, the simulated target sizes cannot be visualized for intermediate (ITA=35  deg) to dark (ITA=−54  deg) skin tones. With DAS beamforming [Fig. 3(b)], targets smaller than 1.75 mm are visible for very light and light skin tones but are difficult to visualize with darker skin tones (ITA≤35  deg) due to the presence of increasing levels of acoustic clutter. With SLSC beamforming [Fig. 3(c)], smaller targets (<1.75  mm) are visible for very light to intermediate skin tones. Although targets ≥2.5  mm can be visualized for the tan (ITA=20  deg) skin tone, target visualization is generally difficult for tan to dark skin tones.

**Fig. 4:**
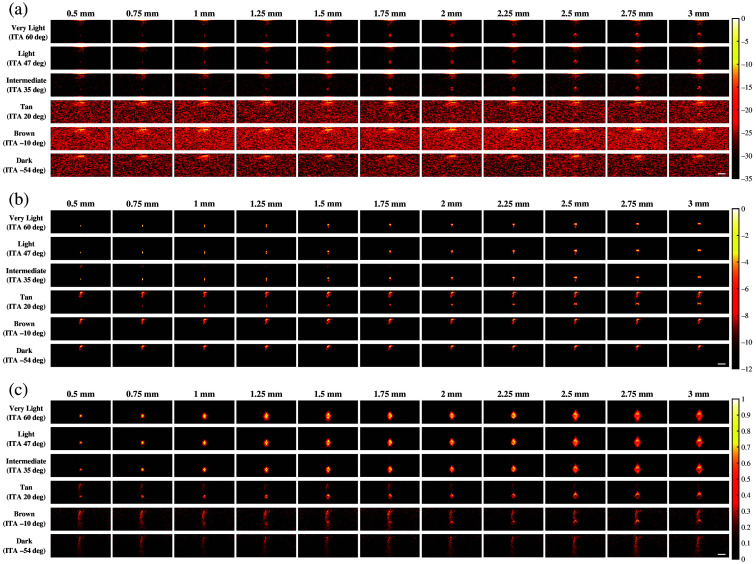
Photoacoustic images created with 800 nm wavelength, multiple skin tones, and (a) FFT, (b) DAS, and (c) SLSC beamforming. The white scale bar represents 5 mm.

Figure 4 shows representative photoacoustic images using 800 nm wavelength and the same three beamformers noted above (i.e., FFT, DAS, and SLSC). With FFT beamforming [Fig. 4(a)], target sizes as small as 0.5 mm are visible with very light and light skin tones, target visualization is more difficult with the intermediate skin tone, and none of the simulated targets are visible with the tan to dark skin tones. With DAS beamforming [Fig. 4(b)], target sizes as small as 0.5 mm are visible for very light to intermediate skin tones, yet they are more difficult to visualize for tan to dark skin tones due to absorption at the skin surface and the associated skin photoacoustic signals and corresponding presence of acoustic clutter. With SLSC beamforming [Fig. 4(c)], the simulated targets are visible with very light to tan skin tones, yet they are difficult to visualize with brown (ITA=−10  deg) and dark (ITA=−54  deg) skin tones.

**Fig. 5:**
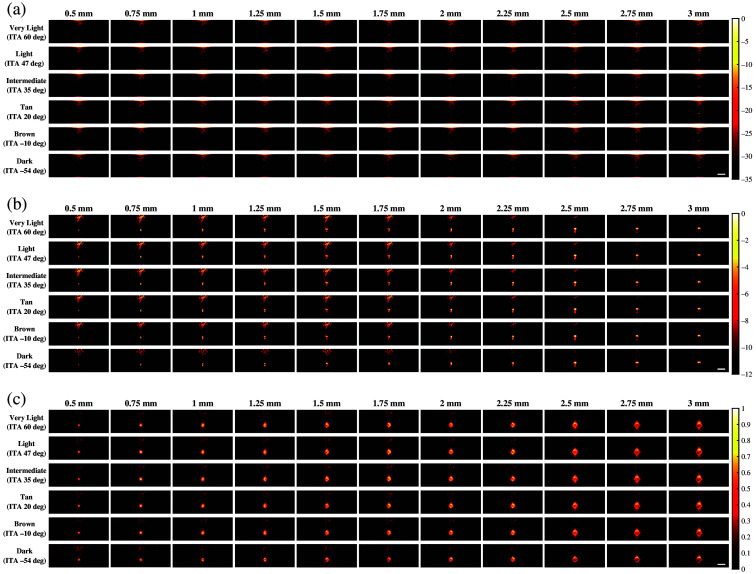
Photoacoustic images created with 1064 nm wavelength, multiple skin tones, and (a) FFT, (b) DAS, and (c) SLSC beamforming. The white scale bar represents 5 mm.

Figure 5 shows representative examples of photoacoustic images using the 1064 nm wavelength and the same three beamformers noted above (i.e., FFT, DAS, and SLSC). With FFT beamforming [Fig. 5(a)], target visualization is generally poor. This is due to increased optical absorption in the initial water layer,^25^ which decreases light fluence into the tissue. With DAS beamforming [Fig. 5(b)], targets ranging from 0.5 to 3 mm can be partially visualized with the dark skin tone. Although the 2 to 2.5 mm targets were better visualized than the smaller or larger targets that were simulated with the very light to dark skin tones, larger targets (2.5 to 3 mm) are generally more visible than smaller targets (≤1.75  mm) for very light to brown skin tones due to acoustic clutter. With SLSC beamforming [Fig. 5(c)], target sizes ranging from 0.5 to 3 mm are visible with the entire range of simulated skin tones.

### 3.2 Target Detectability Metrics

**Fig. 6:**
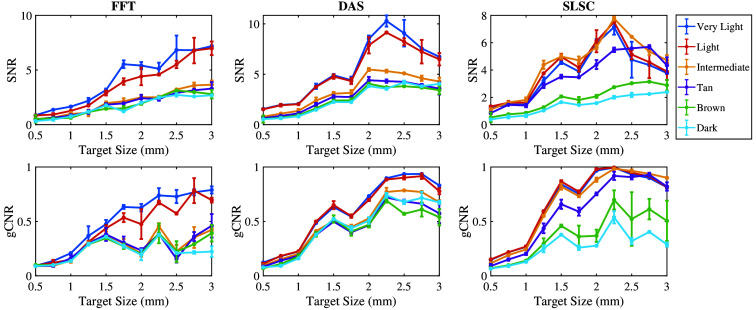
Mean ± one standard deviation of the SNR and gCNR of photoacoustic images created with 757 nm wavelength, per skin tone category, per beamforming method, displayed as functions of the target size.

Figure 6 shows the mean ± one standard deviation of the SNR and gCNR as functions of the target size per skin tone category per beamforming method when imaging with the 757 nm wavelength. With FFT beamforming, the SNR and gCNR generally increase as the target size increases for very light to light skin tones. The SNR and gCNR are lower with intermediate to dark skin tones compared with the SNR and gCNR achieved with lighter skin tones. With DAS beamforming, although the gCNR of targets underlying intermediate to dark skin tones consistently increase as the target size increases, the SNR of targets underlying very light to light skin tones is greater than values achieved with the intermediate to dark skin tones. In this case, the differences in quantitative SNR results are more consistent with qualitative observations of target visibility (when compared with the corresponding gCNR results). With SLSC beamforming, the SNR and gCNR values generally increase as the target size increases in very light to tan skin tones, whereas the magnitude of the increase is lower for brown and dark skin tones compared with that of lighter skin tones. Therefore, very light to tan skin tones are more visible with smaller target sizes with SLSC beamforming.

**Fig. 7:**
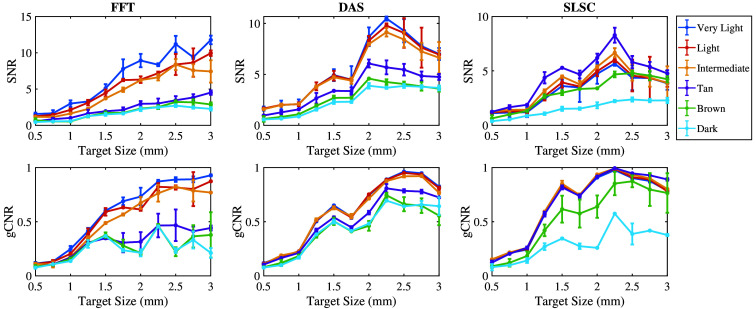
Mean ± one standard deviation of the SNR and gCNR of photoacoustic images created with 800 nm wavelength, per skin tone category, per beamforming method, displayed as functions of the target size.

Figure 7 shows the mean ± one standard deviation of the SNR and gCNR as functions of the target size per skin tone category per beamforming method when imaging with an 800 nm wavelength. With FFT beamforming, the SNR and gCNR generally increase as the target size increases in very light to intermediate skin tones. However, the same rate of increase was not achievable with tan to dark skin tones, which also have lower SNR and gCNR values. With DAS beamforming, the SNR and gCNR of target sizes underlying very light to intermediate skin tones are greater than corresponding values achieved with tan to dark skin tones. With SLSC beamforming, the SNR and gCNR of target sizes underlying very light to tan skin tones are generally greater than values achieved with dark skin tones, whereas brown skin tones have inconsistent quantitative results when compared with the qualitative observations.

**Fig. 8:**
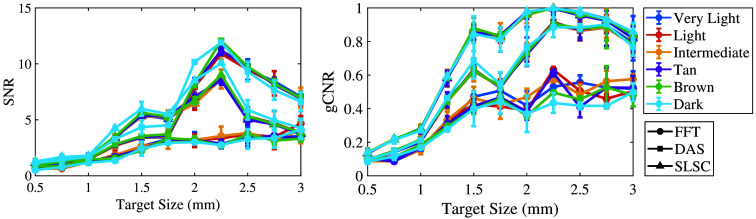
Mean ± one standard deviation of the SNR and gCNR of photoacoustic images created with 1064 nm wavelength, per skin tone category, per beamforming method, displayed as functions of the target size.

Figure 8 shows the mean ± one standard deviation of the SNR and gCNR as functions of the target size per skin tone category for the three beamforming methods (FFT, DAS, and SLSC) when imaging with the 1064 nm wavelength. FFT beamforming produces the lowest SNR and gCNR for most target sizes, which is consistent with the qualitative observation of poor target visibility in corresponding images. With DAS and SLSC beamforming, the SNR is maximized with 2 to 2.5 mm target sizes imaged through very light to dark skin tones, with the greatest gCNR achieved with the 2.25-mm-diameter target. With SLSC beamforming, resulting images generally have the greatest gCNR of the three image formation methods for the simulated target sizes, and there is generally minimal variation among skin tone groups. These quantitative DAS and SLSC observations are consistent with qualitative observations.

### 3.3 Splayed Vessel and Claw Feature Comparisons

**Fig. 9:**
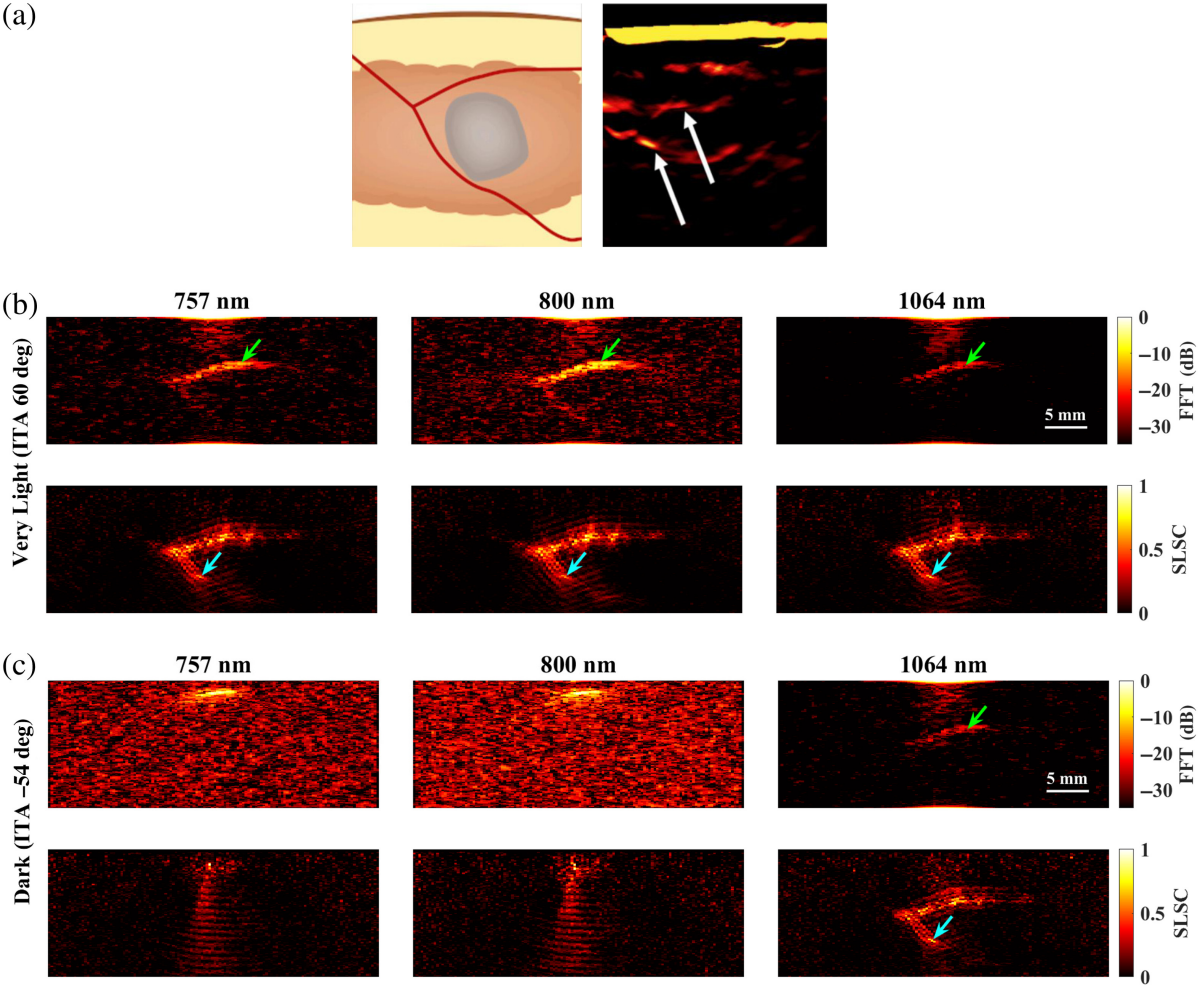
(a) Schematic diagram and corresponding *in vivo* photoacoustic image of a splayed vessel structure visualized with 800 nm wavelength (adapted from Abeyakoon et al.^13^). (b) FFT and SLSC photoacoustic images of a splayed vessel structure for very light (ITA=60  deg) and (c) dark (ITA=−54  deg) skin tones, visualized with 757, 800, and 1064 nm wavelengths. The green arrows indicate a vessel component with a 0.5 mm diameter. The blue arrows indicate vessel components with a 0.75 mm diameter.

Figure 9 shows a schematic diagram and representative photoacoustic image of a splayed vessel structure, created with a standard backprojection algorithm^13^ and 800 nm wavelength [Fig. 9(a)], along with FFT and SLSC photoacoustic images of the simulated splayed vessel structure imaged through very light [Fig. 9(b)] and dark [Fig. 9(c)] skin tones with 757, 800, and 1064 nm wavelengths. The green arrows point to a segment of the proximal vessel that is 0.5 mm in diameter. The corresponding target size simulations predict that this target size is difficult to visualize with FFT reconstruction, 757 and 1064 nm wavelengths, and very light skin tone (ITA=60 deg) and similarly with FFT reconstruction, 1064 nm wavelength, and dark skin tone (ITA=−54 deg). In these three cases, the proximal vessel can be visualized with less clarity compared with the 800-nm FFT result obtained with the very light skin tone. By contrast, the target size simulations predict that 0.5-mm-diameter vessels can be visualized with (1) the very light skin tone, FFT reconstruction, and 800 nm wavelength; (2) the very light skin tone, SLSC beamforming, and 757 to 1064 nm wavelengths; and (3) the dark skin tone, SLSC beamforming, and 1064 nm wavelength. These three cases are consistent with the 0.5-mm-diameter proximal vessel visibility in Fig. 9. The distal vessel (0.5 to 0.75 mm diameter) is difficult to visualize with the FFT reconstruction, yet the blue arrows highlight better visualization of the 0.75-mm-diameter segment of this vessel with SLSC beamforming, using 757 to 1064 nm wavelengths with very light skin tone or a 1064 nm wavelength with dark skin tone (which is comparable to visualization with the very light skin tone).

**Fig. 10:**
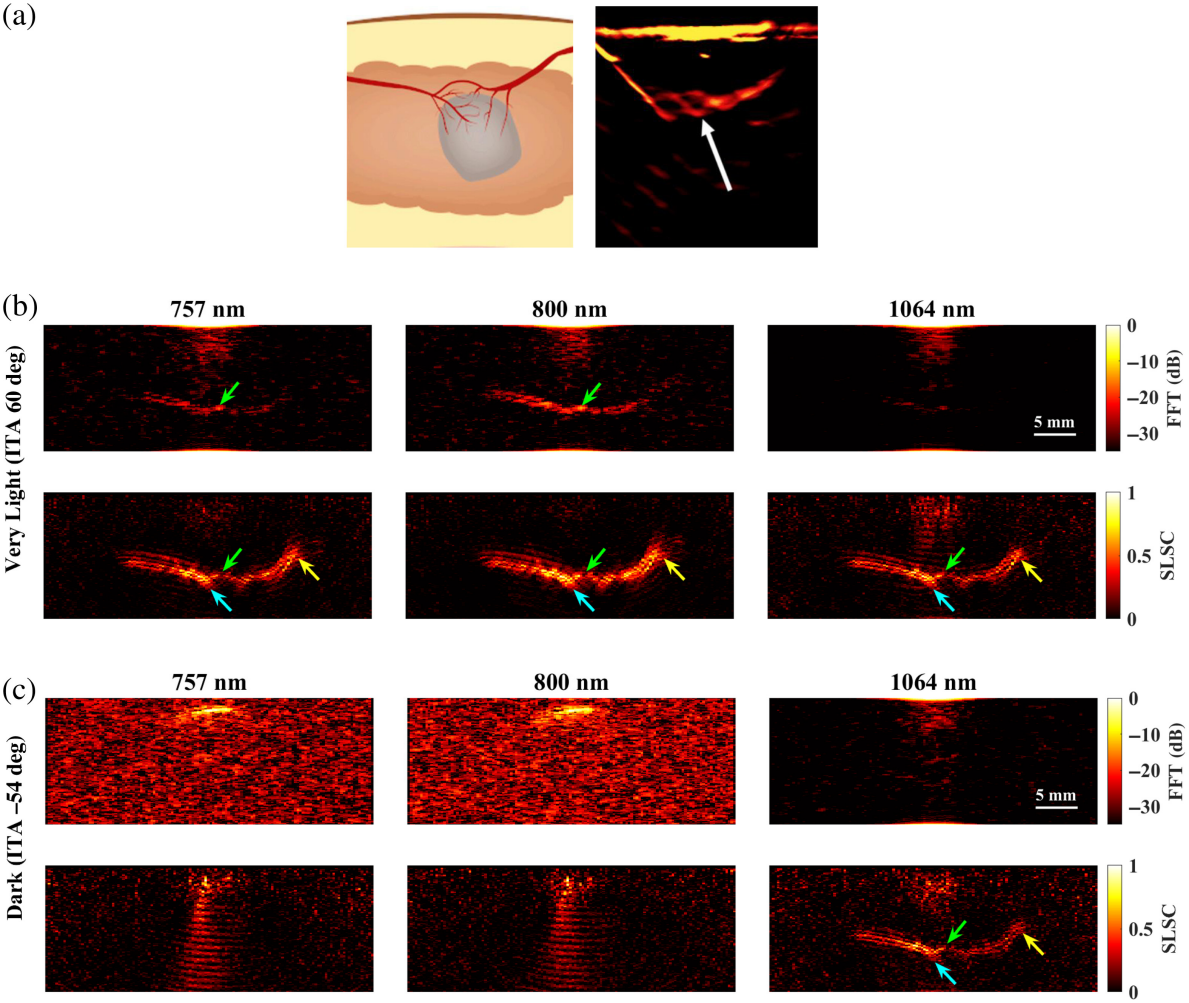
(a) Schematic diagram and corresponding *in vivo* photoacoustic image of a claw structure visualized with 800 nm wavelength (adapted from Abeyakoon et al.^13^). (b) FFT and SLSC photoacoustic images of a simulated claw structure visualized through very light (ITA = 60 deg) and (c) dark (ITA=−54  deg) skin tones, using 757, 800, and 1064 nm wavelengths. The green and yellow arrows point to main vessel components with diameters of 0.5 and 1.25 mm, respectively. The blue arrows highlight a branching vessel component with a diameter ≤0.4  mm that is visible in SLSC images but not visible in corresponding FFT images.

Figure 10 shows a schematic diagram and representative photoacoustic image of a claw structure, created with a standard backprojection algorithm^13^ and 800 nm wavelength [Fig. 10(a)], along with FFT and SLSC photoacoustic images of the simulated claw structure for very light [Fig. 10(b)] and dark [Fig. 10(c)] skin tones, imaged with 757, 800, and 1064 nm wavelengths. The green and yellow arrows indicate components of the main vessel with diameters of 0.5 and 1.25 mm, respectively, which the corresponding target size FFT simulations predict are less visible with 757 to 1064 nm wavelengths and most simulated skin tones. With the very light skin tone (ITA=60  deg) and FFT reconstruction, the main vessel of the claw structure (diameter ranging from 0.5 to 1.25 mm and as small as 0.3 mm in the center) can be visualized with 757 and 800 nm wavelengths, yet is not visible with the 1064-nm wavelength. With SLSC beamforming, 757 to 1064 nm wavelengths, and the very light skin tone, the components of the claw structure highlighted by the green and yellow arrows are clearly visible. The blue arrows indicate areas where the branching vessels (diameter ≤0.4  mm) are partially visible. With the dark skin tone (ITA=−54  deg), visualization of the claw structure is limited to SLSC beamforming and the 1064 nm wavelength, with comparable visualization to the very light skin tone.

### 3.4 Comparisons of Photoacoustic Images to Ground Truth Model Images

**Fig. 11:**
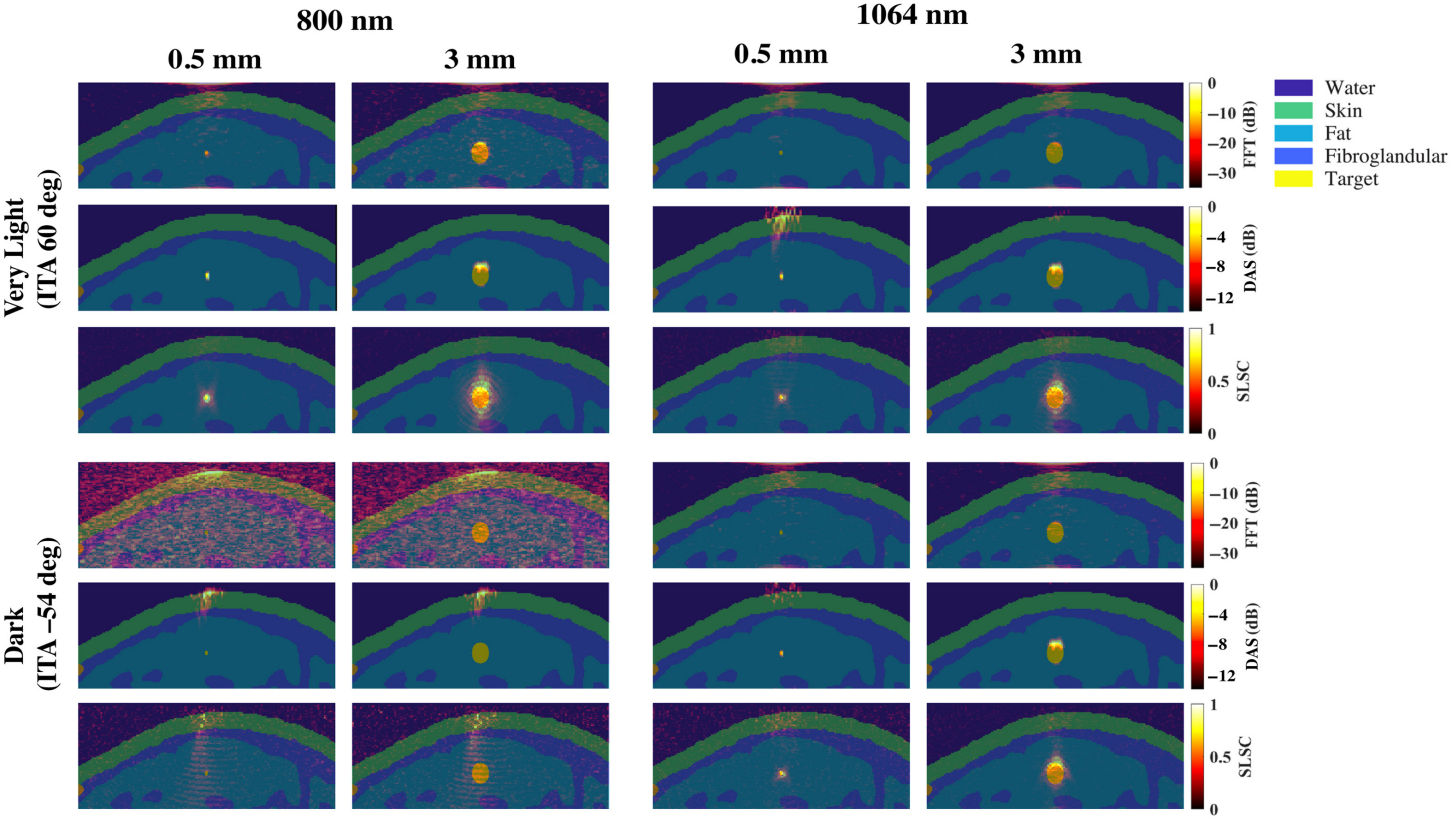
Photoacoustic images from the very light skin tone and dark skin tone results obtained with 800 and 1064 nm wavelengths (Figs. 4 and 5, respectively), overlaid on the corresponding ground truth images from Fig. 1.

It may appear that the targets in the SLSC beamformed images are elongated in the axial direction relative to the amplitude-based images, particularly when viewing Figs. 3–5. To provide more context, Fig. 11 shows examples of model images from Fig. 1 overlaid on photoacoustic images from the minimum (i.e., 0.5 mm) and maximum (i.e., 3 mm) target sizes simulated with 800 and 1064 nm wavelengths and the very light and dark skin tones. In addition to being caused by the axial kernel length required to create SLSC images, the elongated appearance also occurs in the larger targets because the full target is visualized. In particular, the corresponding amplitude-based images (i.e., FFT and DAS images) of these larger targets primarily visualize the proximal region of the larger targets as the remainder of the target suffers from depth-dependent, amplitude-based fluence attenuation. It can also be appreciated from these images that the signal proximal to the target in most images originates from either the skin or water signal in Figs. 3–5.

**Fig. 12:**
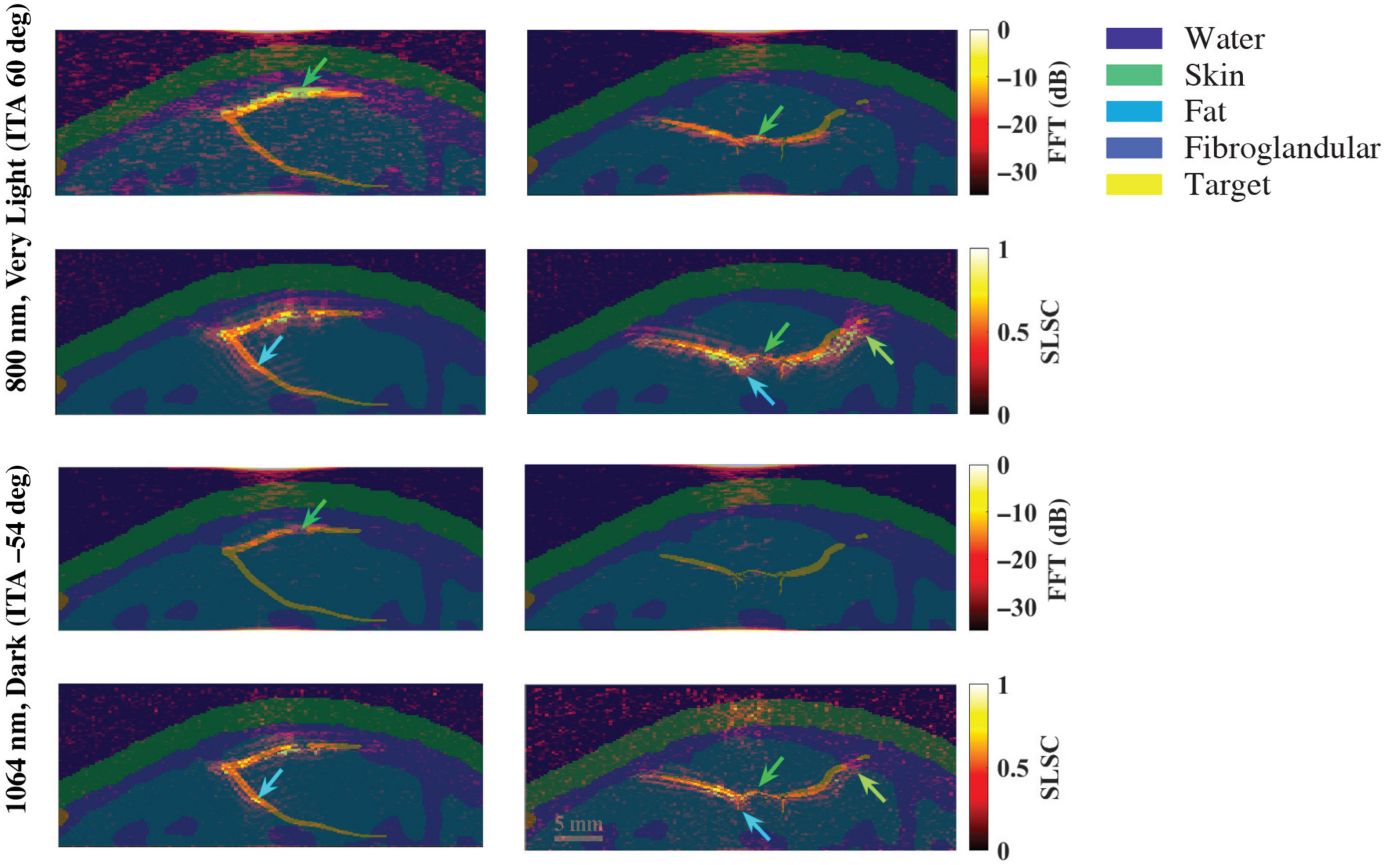
Photoacoustic images from the 800 nm very light skin tone and 1064 nm dark skin tone results in Figs. 9 and 10, overlaid on the corresponding ground truth images from Fig. 2.

Finally, it may appear as if the photoacoustic images in Figs. 9 and 10 are spatially shifted relative to the simulation model. Therefore, Fig. 12 shows simulation model images from Fig. 2 overlaid on corresponding photoacoustic image examples from the 800 nm very light skin tone and the 1064 nm dark skin tone. As opposed to spatial shifts, the overlay format reveals artifacts surrounding the targets in the SLSC images compared with the FFT images, likely due to a combination of the axial correlation kernel length and the presence of coherent artifacts in the simulations. With SLSC beamforming, these types of artifacts are known to decrease with increasing levels of channel noise.^54^^,^^58^

## 4 Discussion

This paper is the first to qualitatively and quantitatively assess the impact of skin tone on target size detectability, followed by demonstrations of multiple combinations of optical wavelengths and beamforming methods that can be used to mitigate the associated skin tone bias, with three key insights. First, smaller target sizes (0.5 to 1.75 mm) are difficult to visualize in general and increasingly difficult to visualize with darker skin tones. Second, the 1064-nm wavelength relieves some of this difficulty when implementing DAS and SLSC beamforming (compared with results obtained with 757 and 800 nm wavelengths). Third, SLSC beamforming, which is a coherence-based approach, further alleviates this difficulty when compared with FFT reconstruction and DAS beamforming, which are both amplitude-based approaches. This insight is consistent with previous reports about SLSC photoacoustic imaging,^25^^,^^33^^–^^36^ which provide foundational evidence that SLSC beamforming outperforms FFT reconstruction when imaging with 750, 810, and 870 nm wavelengths,^34^ and simulated breast imaging was previously demonstrated with 1064 nm and FFT imaging.^25^ With respect to these existing reports, the new and remarkable insight introduced herein is that the SLSC beamformer offers improved target detectability (i.e., gCNR) over FFT and DAS beamforming with a 1064 nm wavelength (Fig. 8).

In most cases, the qualitative observations of Figs. 3–5 are consistent with the quantitative results reported in Figs. 6–8, respectively. For example, the SLSC beamformer improved target detectability compared with the FFT reconstruction when photoacoustic images were acquired with 757 and 800 nm wavelengths in lighter skin tones [shown qualitatively in Figs. 3(c) and 4(c) and quantitatively in Figs. 6 and 7]. In addition, with 1064 nm and DAS and SLSC beamforming, the qualitative (Fig. 5) and quantitative (Fig. 8) variations among skin tones are minimal for each target size, likely due to the greater optical absorption in the water layer at 1064 nm, which decreases optical absorption by the skin and thereby decreases the magnitude of acoustic clutter caused by acoustic wave emission from the skin.^21^^,^^22^^,^^34^ Also, the gCNR values obtained with the 1064-nm wavelength are worse with FFT reconstruction compared with DAS and SLSC imaging in Fig. 8 (which corresponds to the poor visibility of targets with FFT reconstruction in [Fig. 5(a)]), and the acoustic clutter in DAS images of smaller targets [Fig. 5(b)] is reduced with SLSC beamforming [Fig. 5(c)]. Therefore, the combination of SLSC beamforming and the 1064 nm wavelength appears to be the best choice for small target visualization among varying skin tones.

There are a few inconsistencies with the general trends described above. For example, gCNR better supports the qualitative advantages achieved with SLSC beamforming and the 1064 nm wavelength, relative to the SNR, when comparing Figs. 5 and 8. However, the associated gCNR (and SNR) results in Fig. 8 do not support the reduced clutter observed with the dark skin tone DAS images in Fig. 5(b) (relative to the clutter present with lighter skin tones due to optical absorption of water at the 1064 nm wavelength). In addition, with tan to dark skin tones and DAS beamforming, gCNR increases as the target size increases when imaging with 757 nm (Fig. 6) and 800 nm (Fig. 7) wavelengths, although target visualization is qualitatively difficult in Figs. 3(b) and 4(b), respectively. Similarly, with 800 nm and SLSC beamforming [Fig. 4(c)], although the photoacoustic target is less bright when imaging targets underlying the tan skin tone (relative to lighter skin tones), the SNR obtained with the entire range of simulated target sizes and the gCNR obtained with targets ≥2.25  mm are greater with tan skin tones (Fig. 7).

Possible reasons for the above inconsistencies include known differences among the purpose of the SNR and gCNR image quality metrics^55^^,^^56^ and competing differences in the optical absorption of breast tissue^39^ (i.e., 0.004 to $0.005\;{\mathrm{mm}}^{-1}$) relative to that of skin tissue^32^ at the 1064 nm wavelength (i.e., $0.0144\;{\mathrm{mm}}^{-1}$ with the dark skin tone, as opposed to 0.0004 to $0.0035\;{\mathrm{mm}}^{-1}$ with lighter skin tones, based on details in Table 2). The competing optical absorption of the water layer proximal to the skin tissue was also hypothesized to be responsible for the minimal skin absorption in the images created with a 1064 nm wavelength^25^ (i.e., water optical absorption of $0.0144\;{\mathrm{mm}}^{-1}$ versus skin optical absorption ranging 0.0004 to $0.0144\;{\mathrm{mm}}^{-1}$, which is within the range of previously reported values^59^^,^^60^). Additional contributing factors to the inconsistencies noted above include acoustic clutter (e.g., originating from skin, breast tissue, or water optical absorption) that increases the signal in the target ROIs relative to the background ROIs even though no visible target is present, as well as artifacts from the target that increase the overall signal and variance in the background ROI. However, none of these possible reasons for inconsistencies impact the generally optimal combination of SLSC beamforming and 1064 nm wavelength noted above.

It is promising that the benefits observed with 1064 nm and SLSC beamforming translated well from small targets to the splayed vessel (Fig. 9) and claw (Fig. 10) features. In particular, visualization of vessel components as small as 0.5 mm (Fig. 9, green arrows), 0.75 mm (Fig. 9, blue arrows), or 1.25 mm (Fig. 10, green arrows) aligned with target detectability predictions from the corresponding target sizes in Figs. 3–8. In some cases, vessel components with diameters <0.5  mm can be visualized (e.g., blue arrows in Fig. 10), which is promising as these target sizes were not individually simulated in Figs. 3–8. It is additionally promising that, with the 1064-nm wavelength and SLSC beamformer, visualization of the vessel structures underlying the dark skin tone (Figs. 9 and 10) were each comparable to visualization with the very light skin tone.

There are no details about patient skin tone reported by Abeyakoon et al.^13^ when describing the source of the *in vivo* photoacoustic images in Figs. 9 and 10. Therefore, direct comparisons with the simulated 800 nm images for very light and dark skin tones are not possible. In addition to differences between the backprojection algorithm used to generate the *in vivo* images and the three image reconstruction methods used in our simulations (FFT, DAS, and SLSC), there are differences in the light profile and transducer parameters used to generate the *in vivo* images compared with the simulations conducted in this study. There are also likely differences in the level of channel noise, the components of the vessel structures contained within the imaging plane, and post-processing filters that may have been applied to the *in vivo* images after the beamforming step (which our images do not implement). As the literature on *in vivo* photoacoustic images of blood vessels in breast tissue is relatively limited, our goal in simulating the splayed vessel and claw structures was to investigate if our findings regarding SLSC beamforming with the spherical targets translated to more anatomically relevant structures. It is promising that the 1064-nm dark skin tone SLSC photoacoustic images in Figs. 9 and 10 are comparable to the very light skin tone SLSC images created with the 800-nm wavelength (as well as with 757 and 1064 nm wavelengths) and comparable to the *in vivo* photoacoustic images in Figs. 9 and 10, which further supports the use of 1064 nm and SLSC beamforming in a wide range of skin tones. Overall, this comparison to clinical data highlights the importance of including skin tone when reporting results and demonstrates the potential of SLSC beamforming for improving the visualization of blood vessels relevant to breast cancer diagnosis in patients with darker skin tones.

In comparison with previously published *in vivo* forearm results, wherein SLSC beamforming enabled visualization of *in vivo* radial arteries in dark skin tones (ITA range from −53.74 deg to −33.57 deg) with 750, 810, and 870 nm wavelengths,^34^ our simulated results show that targets are not visible with 757 and 800 nm wavelengths and the dark skin tone (ITA range from −54 deg to −33 deg). Given the similar optical properties between 750 and 757 nm wavelengths and between 800 and 810 nm wavelengths,^20^^,^^32^^,^^39^^,^^47^ this discrepancy with the previous report^34^ is likely due to differences in tissue types (e.g., primarily skin and muscle tissue in the forearm as opposed to primarily fatty and fibroglandular tissue in the breast model) and associated differences in optical and acoustic properties.

Although our simulations included multiple tissue components that are known to have varying Grüneisen parameters (e.g., 0.81, 0.16, and 0.12 for subcutaneous fat, blood, and water, respectively^61^), we assumed a constant value across varying tissue types, which could potentially be perceived as a study limitation. However, acceptable values for the Grüneisen parameter of other components of our simulations (e.g., skin and fibroglandular tissue) are not well documented in the literature. The Grüneisen parameter is also temperature-dependent,^61^ has been reported to vary within the same tissue type,^62^^,^^63^ and was previously assumed to be constant in photoacoustic simulations.^39^^,^^64^^,^^65^ As this parameter scales the initial pressure distribution,^66^ incorporating chromophore-specific values is expected to increase the signal from fat tissue relative to components with lower Grüneisen parameters, which could potentially decrease acoustic clutter from skin yet introduce additional clutter from fat.

One limitation of our work is that three wavelengths were investigated, whereas a wider range of wavelengths can possibly be deployed. However, the three investigated wavelengths (i.e., 757, 800, and 1064 nm) correspond to wavelengths utilized in existing clinical photoacoustic imaging systems.^3^^,^^13^^,^^15^^,^^46^ In addition, a wider range of target sizes can potentially be investigated in the future. However, the range of target sizes simulated in this study was based on measurements of diagnostic structures from *in vivo* breast photoacoustic images^13^^–^^17^ and translated well to comparisons with the *in vivo* photoacoustic images in Figs. 9 and 10. Target depth is another variable that can be investigated, although previous work^22^^,^^25^^,^^67^^,^^68^ demonstrated that skin tone influences target detectability at multiple target depths with amplitude-based image reconstruction methods. In particular, deeper targets were difficult to visualize *in vivo* with amplitude-based image reconstruction methods,^22^^,^^25^ this difficulty is further compounded with darker skin tones,^22^^,^^25^ and this compounded difficulty persists as a function of wavelength in controlled phantom^67^ and simulation^68^ experiments. The range of target depths previously investigated includes the target depth of 10 mm from the transducer surface, which was the fixed depth investigated in our study. The coherence-based SLSC beamformer is also known to visualize deeper targets than otherwise possible with amplitude-based beamformers in phantoms^36^^,^^38^^,^^53^ and *in vivo*.^37^ Considering this previous work, we focused our attention on other important parameter combinations—i.e., target size, wavelength, and image reconstruction methods. The results in this paper, when combined with previous findings, add new insights into the detrimental effects of (and potential remedies to) skin tone impacting multiple imaging parameters of interest.

Future testing with phantoms can incorporate synthetic melanosomes that will closely match the absorption coefficient and reduced scattering coefficient of skin.^69^ With a real photoacoustic imaging system, more noise is expected when using low-energy diodes (e.g., light emitting diodes,^22^ pulsed laser diodes,^38^ and continuous wave laser diodes^70^), rather than higher-energy lasers. Although MCXLAB does not support the simulation of specific laser sources or laser energies,^40^ diode lasers are typically commercially available at wavelengths greater than 750 nm,^71^ which aligns with the simulated wavelengths. However, diode sources require extensive signal averaging to compensate for the lower illumination energy, which was previously shown to be resolvable with SLSC beamforming^38^ (assuming the magnitudes of acoustic waves generated after tissue expansion are greater than the noise-equivalent pressure of the transducer^72^). In addition, as the performance of SLSC beamforming is influenced by the presence of channel noise,^33^^,^^54^^,^^58^ future work will investigate the impact of different levels of channel noise on the detectability of targets underlying various skin tones and the relationship to ground truth target sizes.

## 5 Conclusion

The work presented in this paper provides simulation-based evidence that there is a skin tone bias that limits the detectability of small targets (i.e., 0.5 to 3 mm in diameter) underlying darker skin tones when using traditional amplitude-based photoacoustic image reconstruction methods (e.g., FFT and DAS). With SLSC beamforming and transmitted wavelengths of 757 and 800 nm, target visibility remained sufficient with very light to light skin tones (ITA range from 43 deg to 60 deg) and improved with intermediate to tan skin tones (ITA range from 12 deg to 38 deg), relative to amplitude-based FFT and DAS imaging. Adding to these benefits, SLSC beamforming further mitigated the skin tone bias present with brown and dark skin tones (ITA range from –54 deg to 5 deg) when using the 1064 nm wavelength, as demonstrated qualitatively with improved photoacoustic target visibility and quantitatively with increased SNR and gCNR values relative to FFT and DAS images. These findings were further supported when comparing FFT and SLSC images of two realistic vessel structures, as the SLSC images created with a dark skin tone and with a 1064-nm wavelength were comparable to those of a very light skin tone. Differences among skin tone groups were also minimal with 1064 nm per target size per image reconstruction method. This work highlights the promise of the 1064 nm wavelength and SLSC beamforming to address challenges with detecting small targets across a wide range of skin tones. Upon implementation, these parameter choices are expected to improve future designs of next-generation photoacoustic imaging systems.

## Data Availability

Tabulated data to recreate figures that support the major findings of this study are publicly available at https://gitlab.com/pulselab/skin_tone_simulations.^73^ These data were derived from the following software, data, and code available in the public domain: Breast phantom database^39^ k-Wave MATLAB toolbox^48^ Additional data and code related to the paper will be publicly available whenever possible utilizing the above PULSE Lab GitLab repository.^73^
